# *Helicobacter pylori*-induced DNA Methylation as an Epigenetic Modulator of Gastric Cancer: Recent Outcomes and Future Direction

**DOI:** 10.3390/pathogens8010023

**Published:** 2019-02-13

**Authors:** Jibran Sualeh Muhammad, Mohamed Ahmed Eladl, Ghalia Khoder

**Affiliations:** 1 Department of Basic Medical Sciences, College of Medicine, University of Sharjah, Sharjah 27272, UAE; meladl@sharjah.ac.ae; 2 Department of Pharmaceutics and Pharmaceutical Technology, College of Pharmacy, University of Sharjah, Sharjah 27272, UAE; gkhoder@sharjah.ac.ae

**Keywords:** *Helicobacter pylori*, epigenetics, DNA methylation, gastric cancer

## Abstract

Gastric cancer is ranked fifth in cancer list and has the third highest mortality rate. *Helicobacter pylori* is a class I carcinogen and a predominant etiological factor of gastric cancer. *H. pylori* infection may induce carcinogenesis via epigenetic alterations in the promoter region of various genes. *H. pylori* is known to induce hypermethylation-silencing of several tumor suppressor genes in *H. pylori*-infected cancerous and *H. pylori*-infected non-cancerous gastric mucosae. This article presents a review of the published literature mainly from the last year 15 years. The topic focuses on *H. pylori*-induced DNA methylation linked to gastric cancer development. The authors have used MeSH terms “*Helicobacter pylori*” with “epigenetic,” “DNA methylation,” in combination with “gastric inflammation”, gastritis” and “gastric cancer” to search SCOPUS, PubMed, Ovid, and Web of Science databases. The success of epigenetic drugs such as de-methylating agents in the treatment of certain cancers has led towards new prospects that similar approaches could also be applied against gastric cancer. However, it is very important to understand the role of all the genes that have already been linked to *H. pylori*-induced DNA methylation in order to in order to evaluate the potential benefits of epigenetic drugs.

## 1. Introduction

Gastric cancer is amongst the top five most common cancers in the world and is listed as one of the top three leading causes of cancer-related deaths worldwide. Out of these, approximately 50% of deaths are reported in East-Asian countries, like China and Japan [1]. Differences in the disease presentation and consequential late diagnosis hinder clinicians from managing the disease effectively, hence causing higher mortality rates. Chronic *Helicobacter pylori* infection is one of the main causative factors in the development of sporadic gastric cancer. *H. pylori* colonization of the human gastric epithelial cells leads to a precancerous cascade of chronic gastritis, metaplasia, dysplasia, and adenocarcinoma. Atrophic gastritis, metaplasia, and infection by virulent *H. pylori* strains significantly increase the risk of gastric cancer development [2]. *H. pylori* infection is acquired during childhood and then it may persist for life if left untreated. *H. pylori* prevalence and the genotype vary considerably across different regions around the globe [3,4]. *H. pylori* infection damages the gastric mucosa, causing various diseases of the upper gastrointestinal tract such as peptic ulcer, chronic gastritis, gastric cancer and mucosa-associated lymphoid tissue lymphoma [5,6,7,8,9,10,11,12]. 

Pathogenic mechanisms by which *H. pylori* induces gastric cancer have been extensively investigated. However, the exact mechanism of how *H. pylori* infection induces gastric cancer has been a challenging question for decades. However, beyond any doubt, *H. pylori* is involved in gastric carcinogenesis and the World Health Organization (WHO) has classified *H. pylori* as class I carcinogen [13,14]. Numerous epidemiological and molecular studies have shown a decrease in the incidence of gastric cancer in those who received an eradication therapy for *H. pylori*. Several studies associated the carcinogenic properties of *H. pylori* with the presence of specific virulence factors such as CagA, and VacA [14,15].

Genes involved in cancer-related pathways are reported to be are reported to be more frequently affected by epigenetic alterations than by mutations [16]. Cancer development associated with epigenetic alterations could be due to (1) Histone modification: the N-terminal tails of histones may undergo posttranslational covalent modifications such as methylation, acetylation, ubiquitylation, sumoylation or phosphorylation; (2) DNA methylation: which provides a stable gene silencing mechanism that plays an important role in regulating gene expression; (3) Regulation by miRNAs: which can modulate intracellular epigenetic regulatory mechanisms by targeting enzymes responsible for DNA methylation and histone modifications. Furthermore, epigenetic changes in the DNA repair genes can alter the normal processes of the cell cycle, which may lead to increased cell proliferation and initiation of tumorigenesis [17].

*H. pylori* infection induces DNA methylation in the promoter regions of various genes leading to silencing of those genes, facilitating carcinogenesis [18]. Several tumor suppressor genes, protein-coding genes and miRNA’s have been analyzed for methylation status in *H. pylori*-infected noncancerous compared to the cancerous gastric mucosa. Indeed, most of those tumor-suppressive genes were shown to be silenced by *H. pylori*-induced hypermethylation [18,19,20,21,22]. Therefore, it was suggested that accumulation of such aberrant epigenetic field defects in *H. pylori*-infected gastric mucosa predisposes it to gastric cancer development [22,23]. Animal studies have shown that gastric cancer development could be prevented by inhibiting DNA methylation [24].

In this review, we describe a literature survey of the genes affected by *H. pylori*-induced DNA methylation-associated gastric carcinogenesis. We have used MeSH terms “*Helicobacter pylori*” with “epigenetic,” “DNA methylation,” in combination with “gastric inflammation”, gastritis” and “gastric cancer” to search SCOPUS, PubMed, Ovid and Web of Science databases. All the relevant studies selected were included and are described according to specific subheadings mentioned below.

## 2. Role of DNA Methylation in Carcinogenesis

DNA methylation occurs when the cytosine in cytosine-guanine dinucleotides is converted into methyl-cytosine. Hypermethylation of tumor suppressor genes has been strongly linked with cancer initiation and progression. The promoter regions of several tumor suppressor genes and DNA repair genes consist of those Cytosine-Guanine rich regions known as CpG Islands (CGI). Binding of transcription factors to such gene promoter regions is blocked due to their hypermethylation, thus rendering gene silencing or loss of expression. Silencing of tumor suppressor genes would disturb the balance controlling normal cell cycle which may lead to increased cell proliferation and initiation of tumorigenesis. Hypermethylation of previously unmethylated promoter regions of a tumor suppressor gene results in their silencing through inhibition of transcription and inhibition of their ability to suppress abnormal cell proliferation, thus driving the cell through events leading to malignant transformation [25].

## 3. *H. pylori*-induced DNA Methylation in Gastric Cancer

Previous studies highlight a significant connection between *H. pylori*-induced aberrant DNA methylation to gastric cancer development [26]. *H. pylori*-infected Mongolian gerbils were analyzed for DNA methylation levels in their gastric mucosae. Increased methylation was found in the infected animals which correlated with the duration of infection [21,27,28]. *H. pylori* eradication led to a decrease in DNA methylation levels of only a few specific genes [21,22,29], and most of the time high methylation levels persisted even after *H. pylori* eradication. Such hypermethylation was inhibited upon treatment with an immunosuppressive agent. This suggested that *H. pylori*-induced inflammation was important in the induction process of DNA methylation [21]. *H. pylori* infection induces an intense inflammatory response in gastric mucosa, resulting in upregulation of several inflammatory cytokines such as IL-1𝛽, which in turn induces aberrant DNA methylation levels [30]. *H. pylori*-infected macrophages co-incubated with gastric epithelial cells were able to induce *iNOS* expression and increased NO production leading to hypermethylation of *RUNX3* gene in the gastric epithelial cells [31]. Similarly, several *in vivo* studies have shown that *H. pylori*-associated inflammatory responses are mainly involved in DNA methylation leading to gastric carcinogenesis. *H. pylori* eradication in gerbils did not decrease DNA methylation level in the gastric epithelia, but treatment with Cyclosporin-A blocked the DNA methylation induction [28]. In a similar kind of study conducted on gerbils, it was found that using a demethylation agent, such as 5-Aza-2-deoxycytidine, was enough to decrease the level of *H. pylori*-induced DNA methylation to prevent gastric cancer [24]. In addition, clinical studies have also shown that higher levels of DNA methylation were found in some gene promoters, and these methylation levels correlated with the severity of gastric inflammation and precancerous lesions [32]. Gastric biopsies from *H. pylori*-infected patients showing aberrant DNA methylation correlated with a greater risk of developing gastric cancer, thus suggesting that *H. pylori*-induced chronic inflammation could have induced hypermethylation in gastric tissue [33].

Epidemiological data reveal that some of the bacterial virulence factors are associated with gastritis and deregulation of intracellular signaling pathways leading to cancer development. Among those virulence factors, cag pathogenicity island (cagPAI), was associated with an increased level of DNA methylation [34,35]. This cagPAI encodes a type IV secretion system (T4SS) which can transfer macromolecules such as CagA, peptidoglycans and bacterial DNA into the host cell. Infection with CagA positive *H. pylori* induces higher levels of methylation in specific genes compared to CagA negative *H. pylori* [36]. For example, expression of ectopic *cagA* facilitates hypermethylation induced silencing of microRNA let-7 which directly regulates *RAS* expression [37]. 

CagA is strongly associated with the cytotoxic activity of VacA, a virulence factor induced by *H. pylori*. VacA induces multiple cellular activities which disturb endosomal compartments leading to vacuolation of epithelial cells [38]. *H. pylori* strains expressing the combination of both *vacA* and *cagA* are considered most virulent and cause a more severe epithelial damage [39,40] which could be associated with the development of the severe gastric diseases. Till this date, it is unclear whether these virulence factors could also promote epigenetic changes during gastric cancer progression. Further studies are required to explore this question.

## 4. Genes Regulated by *H. pylori*-induced DNA Methylation

Gene expression switching on or off using epigenetic mechanisms, such as DNA methylation, is an essential dynamic regulator of intracellular signaling pathways in the developmental process and disease conditions [41]. Moreover, epigenetic alterations are considered in describing the effect of environmental factors on the human genome, which may enhance the risk of cancer progression [42]. Table 1 summarizes all the genes and their functions, that are linked with *H. pylori*-induced DNA methylation in gastric cancer development (Table 1).

### 4.1. Cell Adhesion Pathway

Studies have reported that *H. pylori*-induced promoter methylation of the *CDH1* gene and eradication of *H. pylori* led to a complete reversal in methylation levels. Relating this methylation to silencing of the *CDH1* gene associated with gastric cancer development, Chan et al. also found promoter methylation of *CDH1* gene in tissue samples obtained from *H. pylori*-infected dyspeptic patients [43]. Perri et al. showed that *CDH1* promoter hypermethylation found in *H. pylori*-infected gastritis patients was reduced after bacterial eradication [22]. *In vitro* experiments by Huang et al. showed that *H. pylori* infection increases *CDH1* promoter methylation in gastric cancer cells. The mechanism involved *H. pylori*-induced upregulation of iNOS production and NO generation via activation of the IL-1β receptor. The total DNA methyltransferase activity was also found to be increased [44].

Vezatin protein found in adherens junctions is subject to hypermethylation-silencing because of the presence of many CGIs in its promoter region. A study analyzing gastric tissue biopsies reported the *VEZT* gene was hypermethylated in *H. pylori*-positive chronic gastritis patients compared with the control non-infected group. Miao et al. found that *H. pylori* infection induces hypermethylation-silencing of *VEZT* in normal gastric epithelial cells [45]. They also reported hypermethylation-silencing of the *VEZT* gene in several other types of cancer, and re-expression of Vezatin reduces the carcinogenic potential of those cancer cells [45]. These findings suggest that Vezatin might serve as a potential target in combating *H. pylori*-associated aberrant methylation and gastric carcinogenesis. Furthermore, *Connexin-32* and *Connexin-43* expression was found reduced in pre-neoplastic gastric lesions linked with *H. pylori* infection when compared to lesions of non-infected patients [46]. *Connexin 32* (*Cx32*) and *Connexin 43* (*Cx43*) are structural components of gap junctions between epithelial cells [47], and it is found down-regulated in precancerous cancerous gastric mucosae. Variation in the expression of *Cx32* and *Cx43* in patients versus controls was associated with high promoter methylation levels. Increased methylation of *Cx32* and *Cx43* promoters was found in the advanced stages from pre-neoplastic to neoplastic lesions in *H. pylori*-infected patients [46].

### 4.2. Cell Cycle Regulation

A cell cycle regulating gene *CDKN2A* encodes p16 (INK4A) protein. This molecule is strongly implicated in G1 phase cell-cycle arrest. Maekita et al. showed that promoter methylation of *CDKN2A* was significantly increased within the gastric mucosae specimens obtained from *H. pylori*-infected patients in comparison to samples obtained from *H. pylori*-uninfected healthy individuals [48]. Perri et al. showed that promoter methylation of *CDKN2A* is linked to *H. pylori*-associated gastric carcinogenesis. Similarities within the levels of promoter methylation of *CDKN2A* were seen in the gastric mucosa in intestinal metaplasia patients. Moreover, after the eradication of *H. pylori* infection methylation levels of *CDKN2A* significantly decreased [22]. 

### 4.3. DNA Mismatch Repair Genes

Perri et al. studied intestinal metaplasia lesions and indicated that the *MHL1* gene. which encodes a DNA repair protein, is hypermethylated in the late stages of *H. pylori*-infected gastric cancer progression. [22]. Sepulveda et al. detected higher methylation levels in the promoter region of O-6-methylguanine DNA methyltransferase (*MGMT*) within the gastritis patients’ gastric mucosae, compared to those of control patients. Significant reduction in the methylation and increase in *MGMT* expression were observed after *H. pylori* eradication. Additionally, in this same study, the authors showed that methylation of *MGMT* was remarkably higher in the samples obtained from patients infected with CagA+ *H. pylori* strains [36]. Alvarez et al. also indicated that hypermethylation of *MHL1* and *MGMT* was detected in biopsies obtained from *H. pylori*-infected chronic gastritis adult patients [49].

### 4.4. Genes Related to Inflammation

Trefoil factor 2 (*TFF-2*) gene encodes TFF2 protein, which is related to wound healing and modulation of gastric inflammation [62,63]. Methylation of *TFF-2* promoter increases during gastric tumor advancement, and chronic *H. pylori* infection is associated with increased promoter methylation of *TFF2* gene in the gastric mucosa [50]. 

Cyclooxygenase-2 (*COX-2*) is a pro-inflammatory enzyme which is crucial in regulating intracellular homeostasis and wound healing in gastric epithelial cells. Therefore, methylation-induced silencing of *COX-2* gene might facilitate the progression of pre-neoplastic lesions. Hypermethylation of *COX-2* has been reported in *H. pylori* infection-associated with gastritis tissue samples, and this hypermethylation was reversed by bacterial eradication [22].

### 4.5. Genes Encoding Transcriptional Factors

*Runt Related Transcription Factor 3* (*RUNX*3) is a tumor suppressor gene and functions as a transcription factor known to regulate the expression of many cancer-related genes, including *p27*, *p53*, and *caspase-3*. *RUNX3* gene is frequently found to be transcriptionally silenced or deleted in cancer tissues [64,65]. Hypermethylation of *RUNX3* promoter was detected in pre-neoplastic gastric lesions, which increased in the proportion showing hypermethylation from chronic atrophic gastritis to gastric cancer [51]. In addition, the *RUNX3* gene was found deactivated via promoter hypermethylation in *H. pylori*-infected gastric epithelial cells [55]. Furthermore, Katayama et al. demonstrated that co-incubation of *H. pylori* and macrophages with gastric cells showed an increase in promoter methylation of *RUNX3* [31]. 

*Forkhead Box D3 (FOXD3)* gene is a representative of the Fox transcription factor family, which is mainly involved in autoimmune disease. Methylated-DNA Capture-Microarray data showed that the *FOXD3* promoter elevated methylation in *H. pylori*-associated gastric tumors in both mice and humans. This increased *FOXD3* methylation was confirmed using methylation-specific PCR with *H. pylori*-positive gastritis tissues compared with uninfected gastric tissue samples. Gastric cancer patients with hypermethylated *FOXD3* survived a shorter time than other patients. *FOXD3* expression was also lower and demethylation treatment increased this expression. *FOXD3*-activated transcription of cell-death-regulating genes *RARB* and *CYFIP2*, and induction of overexpression of *FOXD3* significantly inhibited gastric cancer cell proliferation, facilitating tumor cell apoptosis. [52].

Upstream stimulatory factors (*USF1* and *USF2*) are transcriptional factors known to control gene expression associated with immune responses, cell proliferation, and cell cycle control. They control cell growth and block cMyc/Ras-mediated alteration of primary rat cells [66]. The *in vitro* study of gastric tissue samples obtained from *H. pylori*-infected mice revealed that *USF1* and *USF2* expression was decreased by *H. pylori*-induced hypermethylation of their promoter regions [53]. Higher methylation status was recognized for the transcription factors *GATA4* and *GATA5* in gastric samples from *H. pylori*-infected patients compared to those from non-infected patients. Changes in methylation were mainly detected for the *GATA4* promoter. Also, the gastric tissues with *GATA4* methylation were found to be only from biopsy samples from *H. pylori*-infected patients [54]. In a more recent study, gastric biopsies from *H. pylori*-positive patients exhibited an upregulation of *GATA5* expression compared to that in uninfected patients. However, chronic gastritis biopsies obtained from *H. pylori*-infected patients with hypermethylation of *GATA5* promoter region showed downregulation of *GATA5* gene expression [55]. 

### 4.6. Autophagy-Related Genes

Autophagy is a homeostatic process concerning sequestration of aged cytoplasmic components in autophagosomes, leading to their degradation and then recycling. Autophagy is involved in limiting inflammation and tissue damage, which cause genomic instability. *H. pylori* VacA toxin can disrupt cellular autophagy which enables the survival of autophagy and predisposes to chronic *H. pylori* infection. Individuals with *ATG16L1* 300A polymorphism were found more susceptible to the toxigenic effects of VacA due to a reduced host autophagic response [67]. Microarray analysis of several autophagy-related *(ATG)* genes in *H. pylori*-infected gastric mucosae revealed that 16 genes were upregulated, and 9 genes were downregulated. Out of these 9 genes, *ATG16L1* expression was found significantly inversely correlated with the intragastric density of *H. pylori* in the atrophied gastric mucosa [56]. *MAP1LC3* is another *ATG* gene linked to *H. pylori* infection, and upon *H. pylori* infection, conversion of LC3-I to LC3-II indicates formation of autophagosomes [68]. *MAP1LC3Av1* expression was shown to be silenced due to hypermethylation in *H. pylori*-infected gastric cancer tissues as well as in the adjacent non-cancerous tissues but expression was not seen in *H. pylori*-negative gastric tissues. In addition, transient transfection of *MAP1LC3Av1* showed an increase in gastric epithelial cell proliferation and invasion characteristics. The inactivation of *MAP1LC3Av1* disrupted the autophagy pathway leading to an increase in tumorigenicity of gastric epithelial cells [57].

### 4.7. Tumor Suppressor Genes

Peterson et al. reported that promoters of the *LOX* gene, that encodes a lysyl oxidase enzyme and of the *HRASLS* gene, that encodes *HRAS*-like suppressor proteins, are found highly methylated in patients with gastric mucosae with *H. pylori*-infection as compared to those without infection. They also demonstrated that elevated levels of methylation were detected in the CGIs present in the promoter regions of *THBD*, *HAND1*, and *FLN* in *H. pylori*-positive compared to *H. pylori*-negative individuals [26]. In addition, *APC* is hypermethylated in gastritis associated with *H. pylori* infection, and bacterial elimination reduced the proportion of cases with hypermethylated APC promoters [22]. Furthermore, an elevated methylation in the promoter of the gene encoding p41ARC was detected in *H. pylori*-positive individuals when compared to non-infected individuals. The encoded protein regulates the Arp2/3 complex formation and is required for normal cell migration [43,58]. Tumor suppressor gene *WWOX* encodes WW-domain containing oxidoreductase, which is frequently found to be down-regulated in several cancers. An *in vitro* study demonstrated that *H. pylori*-infected gastric cancer cell lines showed higher methylation of *WWOX*, which was associated with *H. pylori*-induced enhanced expression of *DNMT1* and *DNMT3* [59]. Deregulation of *CYLD*, a tumor suppressor gene, has been found in different types of cancer. Association between *H. pylori* infections with hypermethylation and reduced *CYLD* expression was observed in 10 out of 21 CagA-positive, *H. pylori*-infected tumor samples [60]. *PTEN* is another tumor suppressor gene, which serve important functions in apoptosis, cell cycle progression and cell proliferation. The expression levels of *PTEN* were found significantly decreased in the gastric cancer tissues infected with CagA-positive *H. pylori* compared with gastric cancer tissues infected with CagA-negative *H. pylori*. In addition, the decreased expression of *PTEN* in CagA-positive gastric cancer tissues were associated with increases in its promoter methylation levels [61].

## 5. Conclusions and Future Direction

Sporadic gastric cancer involves multiple genetic and epigenetic alterations, and the disease usually presents in advanced stages. Several studies have revealed that aberrant DNA methylation of tumor suppressor gene promoters likely plays a critical role in gastric carcinogenesis. *H. pylori*-induced hypermethylation silencing of several genes may lead to carcinogenesis in gastric epithelial cells. In this review, we summarized specific genes affected by *H. pylori*-induced methylation and how their expression levels might function as an early indicator of the carcinogenic transformation of non-cancerous mucosae in *H. pylori*-infected gastric cancer patients. 

Several targeted therapies have been developed and are successfully used in different types of cancer. Nevertheless, only a small subset of gastric cancers can be targeted with currently approved novel biological approaches. Epigenetic treatment offers a potential therapy to treat chemo- and radio-resistant tumors; but, due to the limited knowledge of carcinogenic mechanisms, these experimental treatments are still in their infancy. Further studies are required to reveal epigenetic changes as the basis for targeted molecular therapy or novel biomarkers that predict gastric cancer prognosis. We suggest that chemoprevention of gastric cancer induced via *H. pylori*-associated epigenetic changes can be achieved using de-methylating agents. The success of epigenetic drugs in the treatment of certain hematological cancers opened new possibilities that similar approaches can be used to treat solid tumors such as gastric cancer. However, thorough elucidation of cancer-associated molecular background is very important in each individual patient in order to determine who could benefit from administration of such epigenetic drugs. DNA methylation studies may help in differentiating aggressive tumors from relatively benign and slow-growing ones. This may aid in decision making regarding the selection from more or less aggressive treatment modalities.

## Figures and Tables

**Table 1:** Description of the genes linked with *H. pylori*-induced DNA methylation in gastric cancer development.

Gene ID	Gene Function (http://www.genecards.org/)	Reference
*CDH1*	Regulate cell-cell adhesions, mobility, and epithelial cell proliferation	[22,43,44]
*VEZT*	Establishment and maintenance of adherens junctions	[45]
*CX32*	Forms gap junction channels that facilitate the transfer of ions and small molecules between cells	[46,47]
*CX43*		[46,47]
*CDKN2A*	Regulate cell cycle and act as tumor suppressors	[22,48]
*MHL1*	A tumor suppressor gene involved in DNA mismatch repair	[22,49]
*MGMT*	Involved in cellular defense against mutagenesis and alkylating agents	[36,49]
*TFF2*	Stabilizes gastric mucus layer and affects healing of the epithelium	[50]
*COX-2*	Key enzyme in prostaglandin biosynthesis	[22]
*RUNX3*	A transcription factor and functions as a tumor suppressor	[31,51]
*FOXD3*	A transcriptional repressor and/or activator	[52]
*USF1*	A cellular transcription factor	[53]
*USF2*	Transcription factor that binds to a symmetrical DNA sequence	[53]
*GATA4*	DNA binding and chromatin binding transcription factor activity	[54]
*GATA5*		[54,55]
*ATG16L1*	Part of a large protein complex that is necessary for autophagy	[56]
*MAP1LC3A*	Involved in the formation of autophagosomes	[57]
*LOX*	Crosslinking of collagen and elastin, pro-peptide is a tumor suppressor	[50]
*HRASLS*	Exhibits calcium-independent phospholipase activity	[50]
*THBD*	Calcium ion binding and transmembrane signaling receptor activity	[50]
*HAND1*	Transcription factor involved in development and differentiation	[50]
*FLN*	Remodeling of the cytoskeleton, changes cell shape and cell migration	[50]
*p41ARC*	The p41 subunit of Arp2/3 complex that controls actin polymerization	[43,58]
*WWOX*	Acts as a tumor suppressor and plays a role in apoptosis	[59]
*CYLD*	Regulation of cell survival via its effects on NF-κB activation	[60]
*PTEN*	A tumor suppressor, negatively regulates AKT/PKB signaling pathway	[61]
